# Supplementary material for: ProteinDock: A physics-informed layer to improve protein-protein docking reliability
Source: bioRxiv. 2026 Jul 20:2026.07.17.739238. Preprint. [Version 1] doi: 10.64898/2026.07.17.739238 (PMC13420419; doi:10.64898/2026.07.17.739238)
Supplement: Supplement 1 [file media-1.pdf]

Supplementary Information

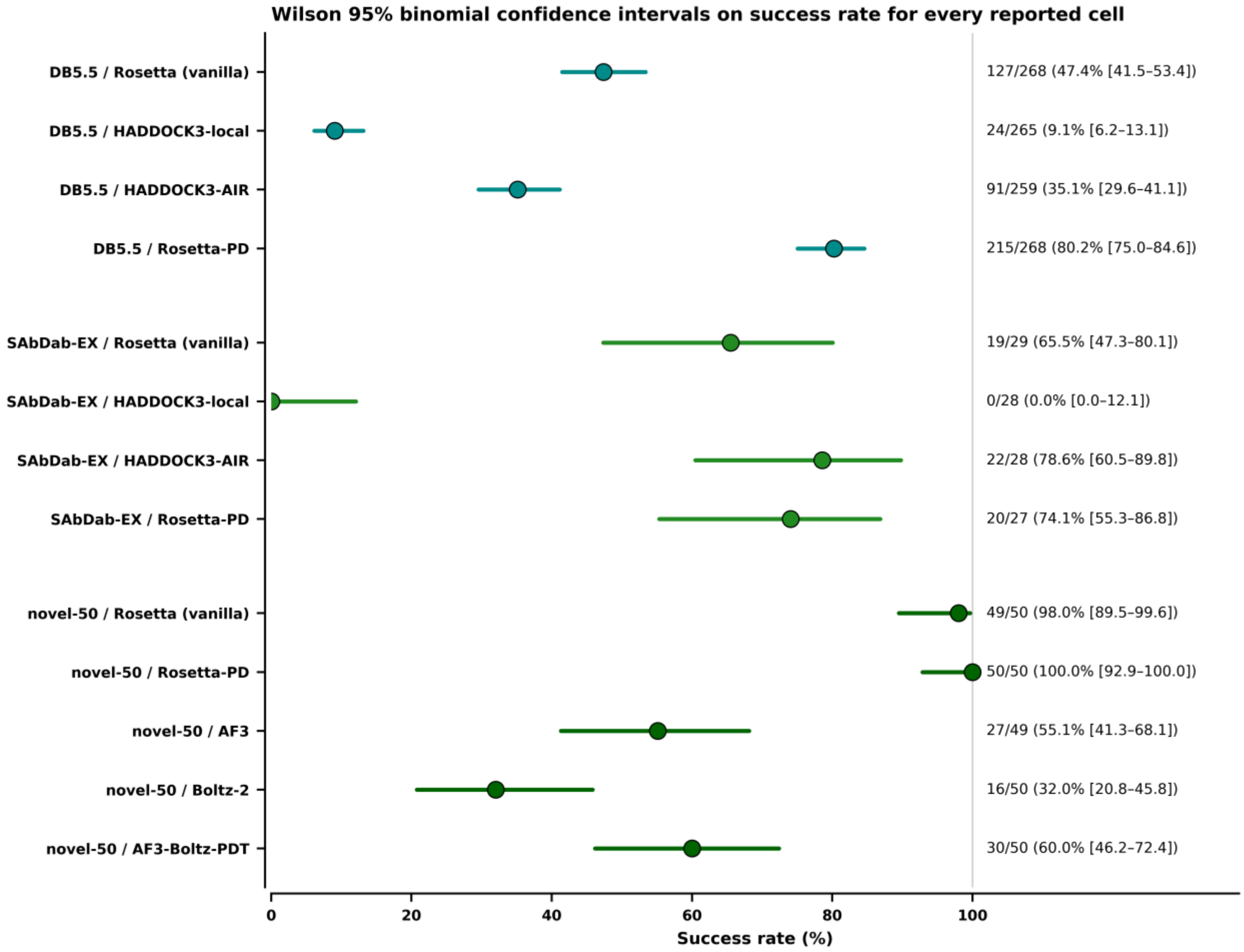

Supplementary Figure 1. Wilson 95% binomial confidence intervals on top-1 success rate for all methods and benchmark cells reported from the main text.

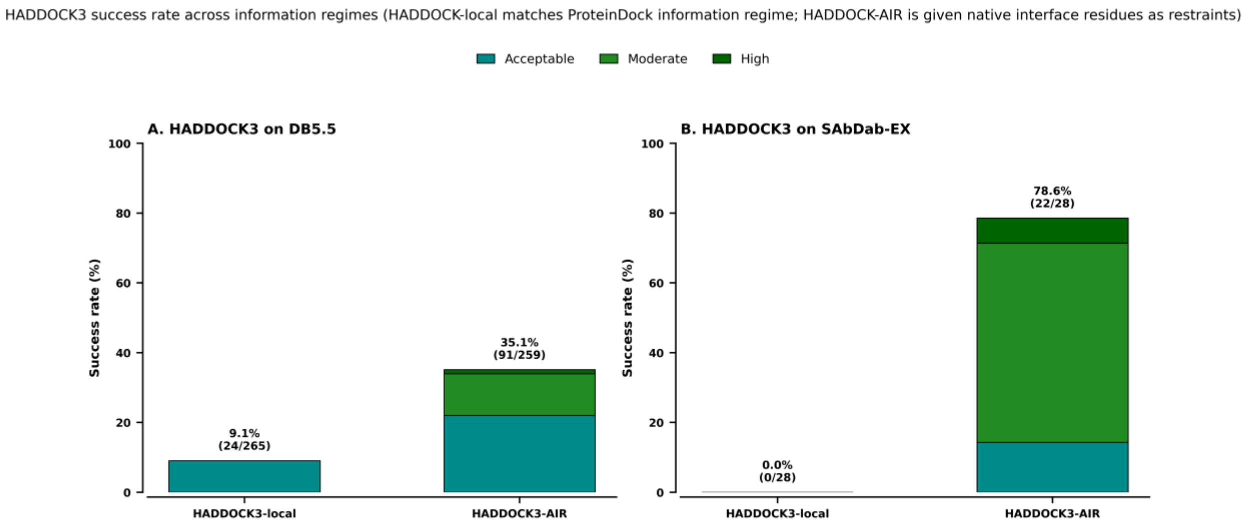

Supplementary Figure 2. HADDOCK3 success rate across HADDOCK3-local and HADDOCK-AIR for DB5.5 and SAbDab-EX.

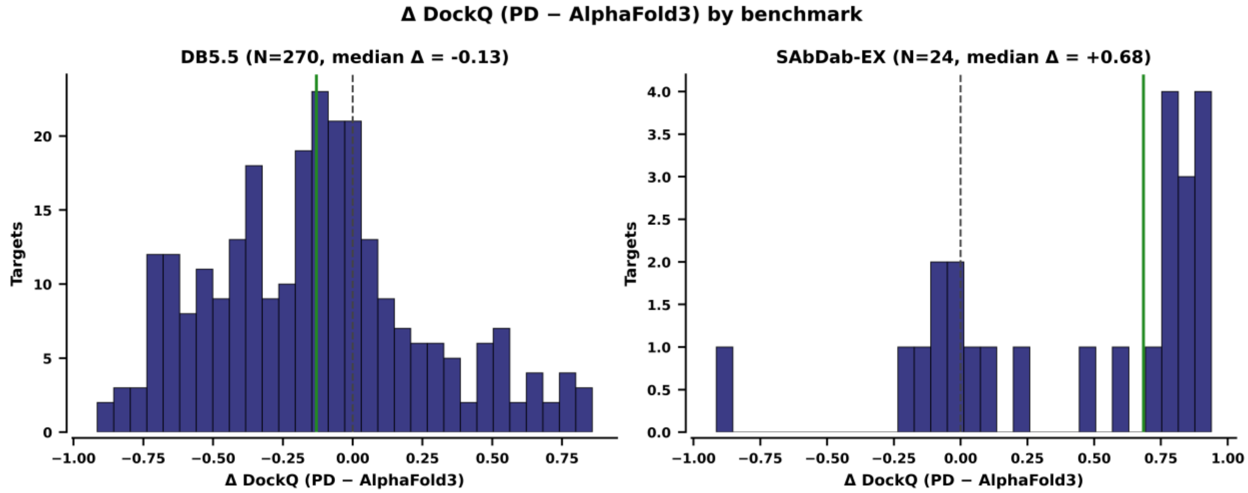

Supplementary Figure 3. Distribution of per-target ∆DockQ (ProteinDock-AlphaFold3) side by side with DB5.5 and SAbDab-EX.

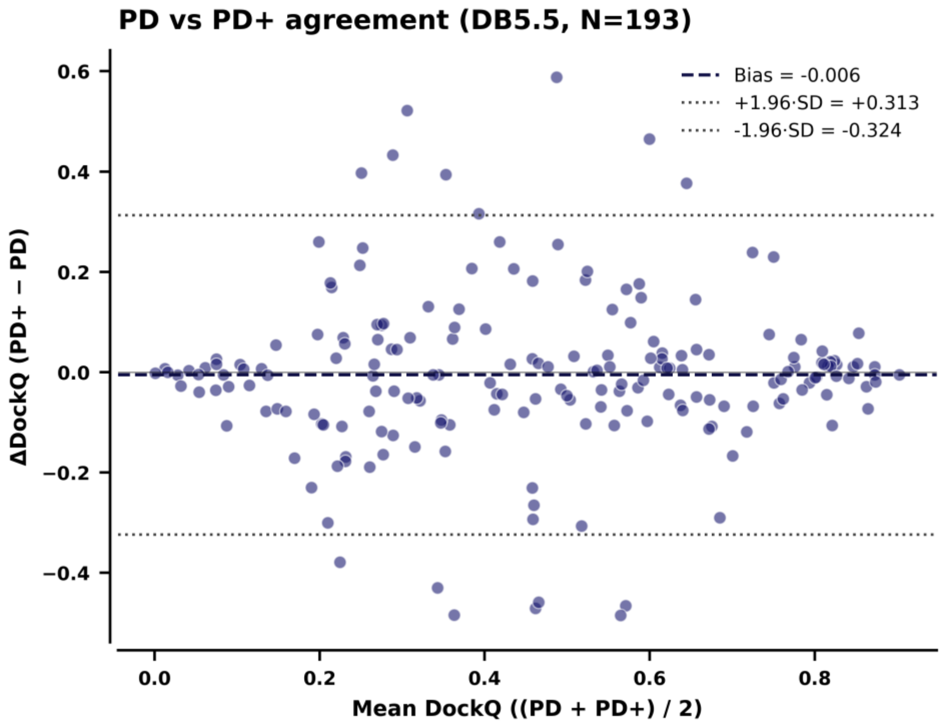

Supplementary Figure 4. Bland-Altman agreement between ProteinDock (PD) and PD+ per-target DockQ on paired DB5.5 targets, displaying mean bias and standard deviation limits.

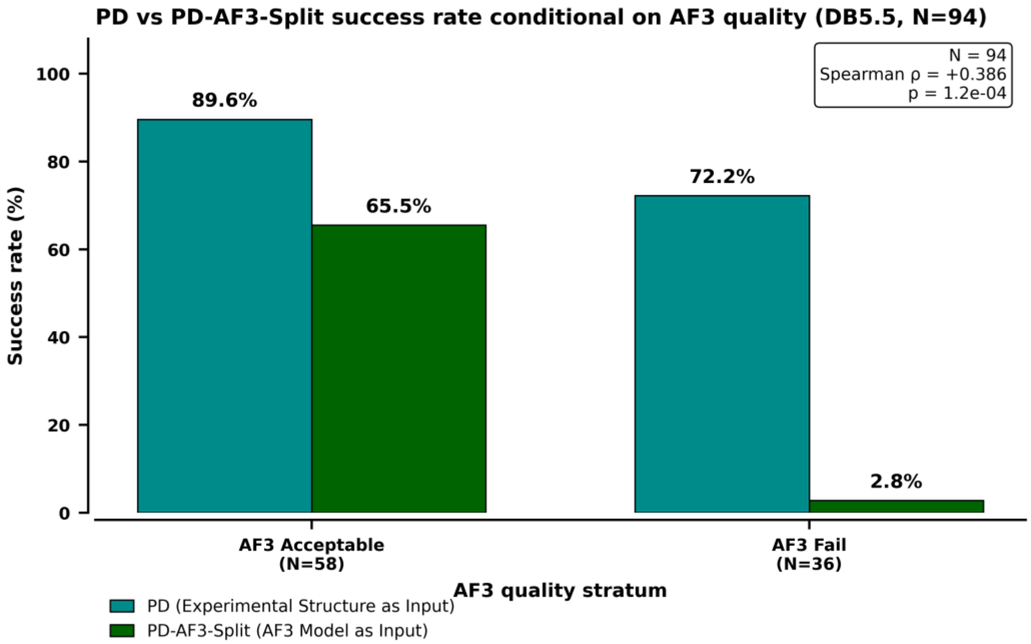

Supplementary Figure 5. ProteinDock success rate conditional on AlphaFold3 pose quality (DB5.5, N=94), comparing PD with PD-AF3-Split (AlphaFold3 model as input).

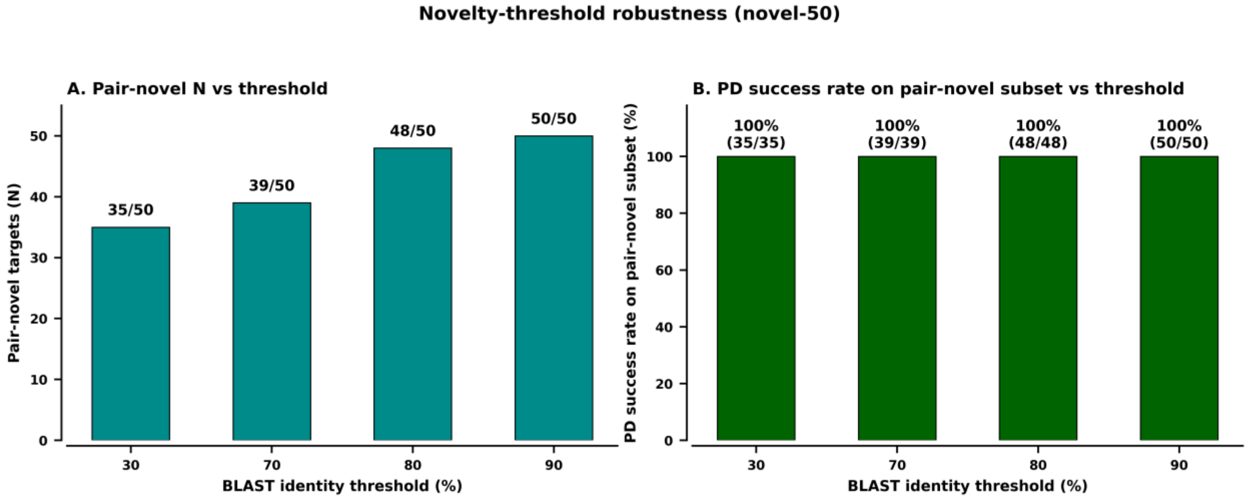

Supplementary Figure 6. Novelty-threshold robustness on novel-50. (A) Number of pair-novel targets vs BLAST chain-identity threshold. (B) ProteinDock top-1 success rate on the pair-novel subset at each threshold.

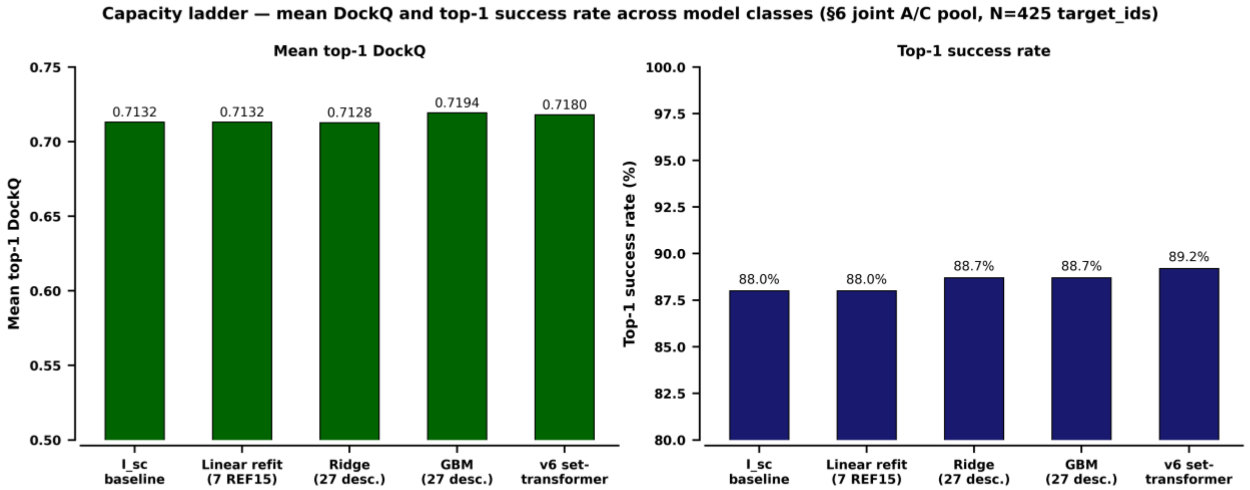

Supplementary Figure 7. Model-capacity ladder across different designs to assess ref2015-scoring capacity.

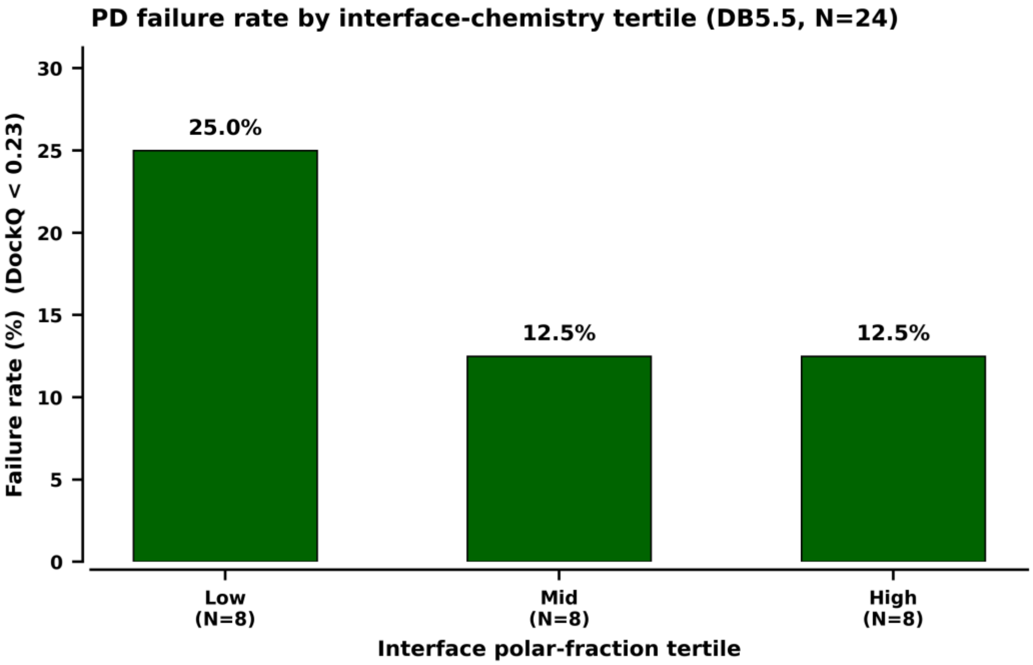

Supplementary Figure 8. PD failure rate (top-1 DockQ < 0.23) on DB5.5 separated into equal-sized tertiles of mean interface polar fractions.

Supplementary Table 1. Dataset curation.

	DB5.5	SAbDab-EX (SAbDab-Expanded)	novel-50
Source	(Docking Benchmark; Vreven et al., 2015)	20 complexes from SAbDab (Structural Antibody Database; Dunbar et. Al, 2014) + 9 complexes recovered from DB5.5	PDB
Version/snapshot	2015 release	October 2024	Depositions 2021-10-01 to 2026-04-15
N	271	29	50
Inclusion	Unbound structures available for both partners	Heterodimeric antibody-antigen pairs	Heterodimeric antibody-antigen pairs with paired H and L chains
Exclusion	——	Complexes containing nucleic acid components	Antibody-antibody pairs; engineered crystal contacts
Resolution	No explicit threshold set	≤ 2.8 Å	≤ 3.0 Å (relaxed from ≤ 2.8 Å to reach N=50; 39 of 50 targets meet ≤ 2.8 Å)
Data Range	1995-2015	——	Post-AlphaFold3 cutoff (2021-09-30)
Overlap	27 of DB5.5's antibody-antigen entries appear in SAbDab-EX	27 of 29 also in DB5.5; 1EZV and 1G9M are SAbDab only	No overlap with SAbDab-EX

Pair-novelty was calculated with *BLAST+ 2.16.0* (blastp –evalue 1e-5) against 660,533 chain sequences from all 185,986 PDB entries from before 2021-09-30. A chain hit was kept if it reached at least 80% of the query length. A binding pair was counted as seen (and omitted from the pair-novel set) if at least one pre-cutoff PDB entry contained both a quality antigen hit and a qualifying antibody-chain hit in the same structure

Supplementary Table 2. Structure preparation by stage.

	DockQ native	Mode 1 input	Mode 2 predictor pose
Waters/ ligands/ ions	Removed	Removed at chain preparation (fix_relabel_renumber.py strips water, ligands, and ions prior to FastRelax	n/a
Chains retained	Only protein chains listed in the benchmark index	Receptor + binder	As given by the predictor
Non-standard amino acids	Mapped to the canonical 20	Removed (ACE/NME caps, hetatoms, modified residues stripped before combining; using only standard 20 amino acids)	n/a
Hydrogens	Not added prior to scoring	Stripped on preprocessing; FastRelax re-adds hydrogens under ref2015 default during pre-relax step	Not added
Chain relabelling	——	Receptor: A; Binder: B . Multi-chain binders (antibody H+L) merged into a single relabeled binder	Receptor: A; Binder: B
Residue Renumbering	——	Reset to sequential intergers per relabeled chain	Renumbered before scoring
Script	fix_relabel_renumber.py	fix_relabel_renumber.py	fix_relabel_renumber.py

Receptor and binder chains were assigned per target with *Rosetta's* '-partners A_B' flag, with antibody H and L chains being specified as belonging to the same binder group with the purpose of maintaining CDR-framework geometry even through pre-relaxation. The system completes chain-labeling and scoring independently for each candidate per run to prevent cache bias in the final output.

Supplementary Table 3. Target counts by analysis.

Analysis	Dataset(s)	N targets	Subset	Why this subset	N decoys
Mode 1 primary	DB5.5	271	All	——	9722 given
Mode 1 primary	SAbDab-EX	29	All	——	nstruct=50 requested; 1,125 delivered
Mode 1 primary	novel-50	50	All	——	nstruct=50 requested; 1,756 delivered
Pair-novelty	novel-50	42	Pair-novel	Absent from pre-cutoff PDB analyzed by BLAST	——
fa_elec sensitivity sweep	novel-50	42	Validation subset	Test best value for fa_elec	——
AF3-refinement runs	novel-50	42	AF3-scorable	AF3 produced a scorable pose	——
Reliability layer	novel-50	50	All	Failures remain in the denominator	25/target (AF3 pool)
Capture radius	novel-50	15	15 targets sampled uniformly across novel-50	——	50 per cell x 60 cells
Capacity sweep pool	novel-50 + SAbDab + DB5.5	75 unique antibody-antigen complex PDBs	novel-50 (50) + SAbDab (18) + DB5.5 recovered (7)	——	13,240 across 425 groups

Supplementary Table 4A. Variant specification (full), mean variants.

Variant	Mode	Input Source	Pre-relax	Sampling	Rerank	Selection	Role
PD	1	Unbound (DB5.5, SAbDab-3), bound-frame monomers (novel-50)	FastRelax, ref2015 default weights, no constraints	nstruct=50, local docking @ fa_elec x 1.5	——	Top-1 by initial I_sc	Baseline docking method
AF3-PD	2	AF3's 25 seed x sample poses	——	None	——	argmin dG_interface	Single-predictor rescoring
AF3-Boltz-PDT	2	AF3 top-1 + Boltz-2 top-1 (single-seed protocol)	——	None	——	argmin with dG_interface, with fallbacks*	Cross-predictor arbitration

*AF3-Boltz-PDT fallback logic: (1) If AF3 and Boltz-2 produced top-1 scorable top-1 poses, take the pose with the lower dG_interface value. (2) If AF3 produces a pose that Rosetta is unable to score, keep AlphaFold3's top-1 pose regardless. (3) If AF3 did not produce any output due to inference crashes, fall back to Boltz-2's top-1 pose.

Supplementary Table 4B. Variant specification (full), ablation variants

Variant	Mode	Input Source	Pre-relax	Sampling	Rerank	Selection	Result (success rate)
PD+	1	Unbound (DB5.5, SAbDab-3), bound-frame monomers (novel-50)	✓	nstruct=50	Top-5 by initial I_sc, re-relaxed under same weights	Top-1 by post-relax I_sc	50/50 (100% acc); High 82%
PD-AF3-Split	1	AF3 pose (top-1 by ranking_Score)	✓	nstruct=50	——	Top-1 by initial I_sc	15/50 (30%)
PD-AF3-Split+	1	AF3 pose (top-1 by ranking_Score)	✓	nstruct=50	Top-5 FastRelax rerank	Top-1 by post-relax I_sc	12/50 (24%)
PD-AF3-Polish	1	AF3 pose (top-1 by ranking_Score)	Coordinate-constrained FastRelax only	None	——	——	13/50 (26%)
PD-AF3-Refine	1	AF3 pose (top-1 by ranking_Score)	——	Tight rigid-bdoy refine, dock_pert 0.5 1.5, nstruct=25	——	Top-1 by I_sc	0/50 (0%)
PD-AF3-Cluster	1	AF3 pose (top-1 by ranking_Score)	——	Cluster into groups, refine each three ways (FastRelax polish, rigid-body refine, and re-docking), rerank, relax top 5	Multi-metric	Top-1 by post-relax I_sc	11/50 (22%)
PD-AF3-Dock	1	AF3 pose (top-1 by ranking_Score)	——	Full-perturbation local docking	Multi-metric	Top-1 by post-relax I_sc	0/0 (Did not run due to killing AF3 poses)

Supplementary Table 5A. Baseline method specifications: AlphaFold3 (Abramson *et al.*, 2024)

Parameter	Value
Release	Public release (Abramson et al., 2024), 2024-11-11 model weights
Mode	Protein-only
Inputs	Receptors and binder amino-acid sequence; multi-chain antibodies supplied as separate H and L chains
MSA	Generated internally from AlphaFold3's bundled database
Template filtering	Filtered to depositions on or before 2021-09-30 (matching training cutoff; verified per-target that retrieved pre-cutoff chain templates did not encode binding geometry
Seed/samples (DB5.5, SAbDab-EX)	1 seed x 5 diffusion sanmples; highest 'ranking_score' sample taken as prediction
Seeds/samples (novel-50)	5 seeds x 5 diffusion samples (25 candidates/target); highest 'ranking_score' sample across the pool taken as prediction
Hardware	1x NVIDIA A100 (40 GB), Ohio Supercomputer Center (Pitzer/Cardinal)
Runtime	~0.5 GPU-hours/target (DB5.5, SAbDab-EX); ~2.5 GPU-hours/target (novel-50, 5 seeds)

Supplementary Table 5B. Baseline method specifications: Boltz-2 (Passaro *et al.,* 2025)

Parameter	Value
Version	V2.2.1 (boltz CLI, pyPI)
Invocation	boltz predict
Seeds	1 random seed per target
Inputs	Receptor and binder amino-acid sequences, YAML manifest
MSA	Use_msa_server: true<br><br>routed to ColabFold MMseqs2 server (Mirdita et al., 2022; uniref30 + colabfold-envdb-202108)
Sampling	--diffusion_samples 5, --recycling_steps 3
Selection	Top-ranked (model_0) sample taken as per-target prediction
Post-processing	Predictions relabeled to chain A (receptor)/ chain B (binder) for DockQ comparison
Hardware	1x NVIDIA A100 (40 GB), Ohio Supercomputer Center (Pitzer/Cardinal)
Runtime	~0.1 GPU-hours/target

Supplementary Table 5C. Baseline method specifications: HADDOCK3 (Giulini *et al.,* 2025)

Parameter	HADDOCK-local	HADDOCK-AIR
Restraints	Chain coordinates only, no AIRs	Native interface residues as ambiguous interface residues (AIRs), extracted from the bound complex
Interface information	Matched to ProteinDock	Maximum (native contacts)
w_desolv	0.0	Default HADDOCK3 weights
Sampling	100 rigid-body with cmrest →<br>40 flex →<br>Top-1 by HADDOCK score	500 rigid-body with AIRs →<br>80 flex →<br>Top-1 by HADDOCK score

Version: HADDOCKv3 v2026.5.0, run on DB5.5. Standard HADDOCK protocol stages were used throughout. Top-1 selection by HADDOCK score.

Supplementary Table 5D. Baseline method specifications: vanilla Rosetta (Leaver-Fay *et al.,* 2011)

Parameter	Value
Definition of "vanilla"	No pre-relax step, no fa_elec reweight
Inputs	Identical to ProteinDock Mode 1
Preprocessing	Nonstandard residues stripped before combining (ACE/NME caps, hetatoms, waters, ligands, modified residues)
Binary	rosetta_scripts.static.linuxgccrelease
Protocol	DockingProtocol (Gray et al., 2003)
Flags	-dock_pert 3 8, -partners A_B, -score:weights ref2015.wts, -nstruct 50 per target
Selection	Top-1 by Rosetta interface score (I_sc) from docking scorefile
Hardware	Intel Xeon Gold 6148 CPU, 4 cores/SLURM array task, Ohio Supercomputer Center (Pitzer/Cardinal)
Runtime	~2 CPU-hours/target

Supplementary Table 6. Data issues and corrections.

Issue	Effect	Cause	Fix	Analyses Affected
1EZV	Wrong antigen-chain assignment during scoring	SAbDab-EX processing	Corrected before evaluation	SAbDab-EX scoring pipeline
1BVK	Empty antibody.pdb, no scorable decoys	Missing source file in original SAbDab curation	Rebuilt complex	Capacity-sweep pool; SAbDab-EX; Mode 1 decoys
Chain-merge/ numbering	DockQ returns iRMS = 999 even on correctly predicted structures	Numbering mismatch between merged binder and separately numbered native H/L	Binder residues renumbered to the union of native H and L indices, applied uniformly	All DockQ scoring
7 misclassified DB5.5 complexes	Antibody-antigen complexes found in the general-PPI pool	Mis-annotation in DB5.5	Recovery by hand and added to the capacity-sweep pool	Capacity-sweep pool
Non-converging structures	Rosetta docking exits with all decoys rejected	Steric clashes in input complex that FastRelax cannot resolve	Standard processing filter	Vanilla Rosetta DB5.5 (3 targets)

Supplementary Table 7. Descriptor definitions.

Name	Count	Members	Used in
Chemistry descriptors	5	Hydrophobic fraction, polar fraction, interface size (residues), charged residues, salt bridges	Antibody vs. non-antibody interface comparison, Mann-Whitney U (below)
Single-parameter physics candidates	14	Interface size (residues); interface size (heavy atoms); contact count (4.5 Å heavy-atom); contact count (5 Å heavy-atom); sum of Voronoi contact areas; number of hydrogen bonds; number of salt bridges; number of aromatic-aromatic contacts; number of hydrophobic-hydrophobic contacts; number of charged-charged contacts; per-CDR contact counts (H1, H2, H3 aggregated); per-CDR contact fractions (H1, H2, H3 aggregated); packing density (Voronoi cell fraction); clash count (< 3 Å heavy-atom)	Calibration-robustness (I_sc + α· X corrections)
ref2015 energy terms	7	fa_atr, fa_rep, fa_sol, fa_elec, hbond_sc, hbond_bb_sc, lk_ball_wtd	Linear refit; capacity sweep
Interface geometry features	27	Interaction counts within 4.5 Å (charged–charged, aromatic–aromatic, hydrophobic–hydrophobic, H-bond donor–acceptor, salt-bridge); Voronoi-style distance-weighted contact areas; per-CDR contact counts and fractions across all six CDR loops; clash counts; packing density	Capacity sweep, per-decoy
Target context features	5	Receptor size (residues); binder size (residues); interface class (Ab-Ag flag from index); antigen chain count; per-target decoy pool size	Capacity sweep, per-target

Supplementary Methods 1: *Capture-radius characterization*

We perturbed 15 novel-50 targets from their native bound state across four different magnitudes: proximal (2 Å, 5°), near (5 Å, 10°), mid-range (8 Å, 15°), and far (12 Å, 20°), with 60 target-magnitude cells. Each perturbed input was run through the full ProteinDock protocol, and the final outputs were classified into the four CAPRI bins. We used one seed per cell due to the ability of Rosetta's DockingProtocol to sample randomly at each starting pose, already providing the variance an extra seed would otherwise add.

Supplementary Methods 2: *Capacity sweep, full specifications*

The capacity sweep was completed on the pool described in **Supplementary Table 3** under 5-fold PDB-disjoint cross-validation (descriptors in **Supplementary Table 7**).

Model Class	Free Parameters	Training
Single-parameter physics correction, I_sc + α· X	1	α tuned per fold, grid search
Linear ref2015 weight refit	7	Pairewise margin-rank loss
Conventional regressors (27 variants)	10^2-10^4	sklearn defaults
Set-transformer ensemble, 15 seeds	10^5	AdamW, cosine annealing, early stopping*

*Set-transformer architecture: 2-layer encoder, 4 heads, d_model= 96, dropout= 0.25, sigmoid DockQ head. Trained with 1:1 MSE + LambdaRank pairwise loss, AdamW (lr=1 x 10^{-3}), cosine annealing over 120 epochs, gradient clipping at 1.0, early stopping (patience 15) on a 20% internal validation split.
